# Ectopic Adrenocorticotropic Hormone-Producing Metastatic Gastrinoma: A Case Report

**DOI:** 10.7759/cureus.45329

**Published:** 2023-09-15

**Authors:** Shrikant Tamhane, Lakshmi P Menon, Dinesh Edem, Pranjali Sharma, Jhansi Maradana

**Affiliations:** 1 Endocrinology, Diabetes and Metabolism, Baptist Health, North Little Rock, USA; 2 Endocrinology, Diabetes and Metabolism, University of Arkansas for Medical Sciences, Little Rock, USA; 3 Endocrinology, Parkview Medical Center, Pueblo, USA; 4 Endocrinology, Diabetes and Metabolism, Mass General Brigham Wentworth-Douglas Hospital, Dover, USA

**Keywords:** somatostatin analogs, capecitabine and temozolomide, ketoconazole, pancreatic neuroendocrine tumor, ectopic cushing syndrome, metastatic gastrinoma, gastrinoma

## Abstract

Pancreatic neuroendocrine tumors secreting gastrin and adrenocorticotropic hormone (ACTH) are rare. The presentation of the cases can be varied, making the diagnosis challenging and often delayed. Here, we present a patient who presented with severe hypokalemia and was found to have ectopic Cushing’s syndrome. An abdominal CT scan showed a pancreatic lesion with metastatic liver disease. A biopsy of the liver lesion confirmed a metastatic neuroendocrine tumor. The final diagnosis was ectopic ACTH-producing metastatic gastrinoma. Twenty-four-hour urinary cortisol was significantly elevated at 9,790 mcg/24 hours. The excess hormonal secretion was successfully treated with ketoconazole and somatostatin analogs. She was further started on chemotherapy with capecitabine plus temozolomide, which has become the preferred chemotherapy treatment after the results of the recently completed trial. She also received Y90 therapy for metastatic liver disease. The prognosis of metastatic pancreatic neuroendocrine tumors is poor. Multidisciplinary combined therapies can help control disease and improve prognosis. We present an 18-month-long patient follow-up and a literature review of ectopic ACTH-producing metastatic gastrinomas.

## Introduction

Neuroendocrine neoplasms (NENs) are rare neoplasms arising from neuroendocrine cells. Gastrinomas, secreting gastrin, are one of the most common functional pancreatic neuroendocrine tumors (pNETs) [1]. If not diagnosed early, it can lead to advanced disease and poor prognosis in many cases. The secretion of gastrin and adrenocorticotropic hormone (ACTH) by a neuroendocrine tumor is relatively rare. The incidence of Cushing syndrome (CS) affects less than 1% of patients with Zollinger-Ellison syndrome (ZES) [2]. Its clinical presentation varies and tends to be misdiagnosed. Liver metastasis and ACTH production in gastrinomas are associated with a worse prognosis [3]. Treatment is multidisciplinary and aimed at decreasing hormonal secretion and controlling tumor growth. Experience with systemic chemotherapy for metastatic gastrinoma with/without ACTH production is limited. We present a case of ectopic ACTH production from a metastatic gastrinoma with an 18-month-long treatment course. We discuss the challenges for early diagnosis, management of hormonal over-secretion, and tumor growth management. We will also provide a literature review on ectopic ACTH-producing metastatic gastrinomas.

## Case presentation

A pleasant 61-year-old female initially presented to her primary care physician (PCP) with symptoms of fatigue and generalized swelling. She was doing well at her last PCP visit, about five months ago, which was a routine annual visit. Over the last three to four months, she noted worsening symptoms of fatigue, generalized swelling, 6-7 lb weight gain, weakness, lately struggling to walk up two flights of stairs, and mild worsening of her acid reflux symptoms. She has a past medical history of hypertension, treated with hydrochlorothiazide and gastroesophageal reflux disease (GERD), diagnosed about five years ago. She was initially started on H2 blocker, famotidine 40 mg bid for GERD, and later switched to proton pump inhibitor (PPI), omeprazole 20 mg daily.

Her examination on presentation showed 2+ lower extremity edema. She had a chemistry panel done and was referred to cardiology for edema and decreased exercise tolerance. Helicobacter pylori testing was ordered given her worsening acid reflux symptoms despite the use of a PPI. Initial lab work revealed hypokalemia 2.7 mmol/L (3.6-5.2 mmol/L), random blood sugar 224 mg/dl, and elevated ALT 79 U/L (7-56 U/L). Her PCP recommended starting potassium chloride 20 mEq bid and ordered HbA1c, liver ultrasound, and hepatitis panel. HbA1c was elevated at 7.3%, repeated potassium level was 2.6 mmol/L, and the right upper quadrant ultrasound revealed several lesions in the liver suspicious for metastatic disease.

**Figure 1:**
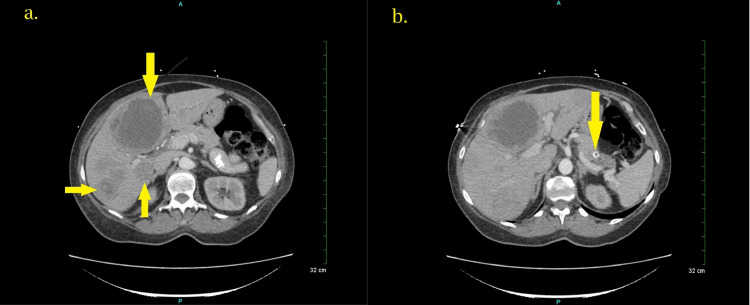
CT scan images of the abdomen with contrast: Multiple hepatic lesions measuring up to 8.2 cm are seen in the image on the left (a), and a 9 mm nodule is identified in the pancreatic tail with a rim seen in the image on the right (b).

She was admitted to the hospital for severe hypokalemia and a liver biopsy. On admission, her potassium level was 1.9 m/L. She was aggressively treated for hypokalemia with potassium replacement (required more than 200 mEq of potassium supplementation daily, amiloride, and spironolactone). With severe hypokalemia and clinical presentation of generalized edema, CS was considered one of the differentials. Labs showed significantly elevated cortisol at 126.1 mcg/dl (2.3-19.4 mcg/dl), ACTH was elevated at 272 pg/ml (7.2-63.3 pg/ml), and non-suppressed dehydroepiandrosterone sulfate (DHEAS) at 113 mcg/dl (29.4-220.5 mcg/dl). Diagnosis of ACTH-dependent CS was made. She had an abdominal CT, which revealed multiple hepatic lesions suggesting primary hepatocellular carcinoma versus hepatic metastases. Additionally, a 9-mm pancreatic nodule within the distal pancreatic tail was noted (Figure 1).

She underwent a biopsy of the liver nodule, which was positive for a neuroendocrine tumor. Given the staining with CDX2 and the lack of reactivity with TTF-1, the site of origin was most likely the GI tract. The final pathology diagnosis was malignant NET, grade 2, Ki-67 10.05% positive. With the lesion in the pancreas and liver biopsy positive for neuroendocrine tumor, a diagnosis of pNET with liver metastasis was made. She had a pituitary-dedicated brain MRI, which showed a normal pituitary and no other significant abnormal findings. On further questioning, she also mentioned that she had diarrhea, which had improved after starting acid reflux medication. Additional labs revealed that the fasting gastrin level was significantly elevated at 1,065 pg/ml (<100 pg/ml), elevated chromogranin A at 1,149 ng/ml (<311 ng/ml), pancreatic polypeptide of 126 pg/ml (56-480 pg/ml), VIP 50 of pg/ml (<78 pg/ml), insulin of 5.1 uIU/mL (<19.6 uIU/ml), prolactin of 14.7 ng/ml (<25 mg/ml), PTH of 34 pg/ml (10-65 pg/ml), and serum calcium within normal limits. Additionally, 24-hour urinary cortisol was significantly elevated at 9,790 mcg/24 hours (4-45 mcg/24 hours). 

The concern was her significant hypercortisolemia and the need to rapidly control it to reduce morbidity/mortality. Metyrapone and mitotane were unavailable in the hospital pharmacy and would have needed more time to procure. She was started on ketoconazole at a high dose of 400 mg orally, three times a day. Liver function tests were closely monitored, and they remained stable. She was also started on octreotide 150 mcg subcutaneously, three times daily. Surgery was consulted for the potential need for bilateral adrenalectomy if medical therapy was not adequate or not tolerated. Cortisol level at the time of diagnosis was 126.1 mcg/dl; after starting ketoconazole and octreotide, cortisol level on day one was 89.1 mcg/dl, on day two was 61.5 mcg/dl, on day three was 53.5 mcg/dl, on day four was 30.2 mcg/dl, and on day five was 18.1 mcg/dl. She responded well with decreasing cortisol levels and decreased requirements of potassium supplements. She initially required close to 200 mEq/day of potassium chloride; however, at the time of discharge, she did not need any potassium supplements and was off spironolactone and amiloride, and her cortisol level was less than 20. She had a new onset of diabetes contributed by significant hypercortisolemia; she was initially treated with insulin to control her blood sugar levels and was discharged on low-dose basal and prandial insulin. At the time of discharge from the hospital, she was symptomatically better.

In the outpatient setting, she is being followed by endocrinology and oncology. She continues on ketoconazole; the dose has been titrated to maintain eucortisolemia, and the latest dose is 100 mg, two times a day. Octreotide was discontinued, and she was started on once-a-month lanreotide. She was started on capecitabine and temozolomide chemotherapy. HbA1c level decreased from 7.8% initially using insulin therapy to 5.5% in six months; insulin was subsequently discontinued, and she is currently taking metformin 500 mg XR twice daily. Her blood sugar continues to be well controlled. Her generalized edema and other symptoms have subsided. Her genetic testing for multiple endocrine neoplasia type 1 (MEN 1) was negative. Her initial gastrin level, which was significantly elevated at 1,065 pg/ml (<100), decreased to 280 pg/mL in six months. Serum ACTH and cortisol levels have been in the normal range. Her latest ACTH was 30 pg/ml, AM cortisol was 10.2 mcg/dl, and DHEAS was 10 mcg/dl. Further, 24-hour urinary cortisol, which initially was 9,790 mcg/24 hours, decreased to 13.9 mcg/24 hours in six months.

She also received Y90 treatment (image-guided hepatic arterial radioembolization). She continues on PPIs, ketoconazole, lanreotide, and chemotherapy. Her latest repeat imaging at her oncology office showed a decrease/stability in the hepatic lesion size for most lesions. She has been stable for nearly 18 months without serious complications.

## Discussion

Neuroendocrine neoplasms (NENs) are rare. They arise from neuroendocrine cells and include both benign and malignant tumors. Gastroenteropancreatic NENs are NENs found in the gastrointestinal system and can be well-differentiated neuroendocrine tumors (NETs) or poorly differentiated neuroendocrine carcinomas (NECs). The pNETs that produce hormones (insulin, gastrin, ACTH, CRH, growth hormone, parathyroid hormone, and calcitonin) are called functional NETs. They also produce other vasoactive substances such as chromogranin A, pancreastatin, and pancreatic polypeptide.

Gastrinomas, which secrete gastrin and cause ZES, are one of the most common functional pNETs. Specifically, 20-60% of gastrinomas arise in the pancreas, and 60-90% are malignant [1]. The annual incidence of gastrinomas is 0.5-21.5 per million population [4]. Additionally, 13-53% (mean 34%) of pancreatic gastrinomas have metastasized to the liver at the time of diagnosis, which is one of the determinants of long-term survival and hence has a worse prognosis [1]. In our patient, the tumor had metastasized to the liver at the time of diagnosis.

The diagnosis of ZES can be challenging, and there could be a lag of a few years from the onset of symptoms until the diagnosis of gastrinoma is made. The average time from onset of symptoms to diagnosis of gastrinoma is about six years [5]. One of the reasons is that the symptoms (peptic ulcer disease, heartburn, diarrhea, weight loss, and acid hypersecretion complications such as bleeding, stricture, and perforation) can be nonspecific and masked by PPI use, which is likely what happened in our patient. She had symptoms of acid reflux and diarrhea about five years ago and was initially treated with H2 blockers and later switched to PPI with an improvement of her symptoms. The diagnosis of gastrinoma only came about after the workup detected the consequences of ectopic ACTH hypersecretion.

Moreover, 20-25% of gastrinomas are associated with MEN 1 [4]. MEN 1 testing should be considered, especially in individuals with a family history of MEN 1, young adults, suspicious clinical or laboratory findings (e.g., renal colic or nephrolithiasis, history of hypercalcemia, elevated prolactin), or multiple MEN1 tumor types (parathyroid gland, anterior pituitary, and enteropancreatic). In our patient, MEN 1 genetic testing was negative.

Ectopic ACTH production by an islet cell tumor of the pancreas is a relatively rare cause of hypercortisolism, accounting for less than 1% of all patients with CS [6]. The excess cortisol production in these patients results in high morbidity and mortality seen. Achieving control of excessive cortisol secretion is crucial in the management of patients. Diagnosis may be challenging as, with the relatively short duration of exposure to a high level of cortisol secondary to ACTH-dependent CS from an ectopic source, patients may not develop all the clinical features of cortisol excess. In the series of 90 patients from NIH with ectopic ACTH secretion, 74% of patients were hypokalemic [7]. The presentation can be varied, such as weight gain and loss, menstrual irregularities, fractures, cognitive and psychiatric disorders, hypertension, elevated blood sugars, infections, weakness, and bruising. Physicians of many specialties often evaluated these patients before the clinical diagnosis was entertained. Once considered, urinary free cortisols (UFCs) were more than 10 times the upper end of normal in more than half of the patients [7]. Our patient presented with severe hypokalemia, weight gain, weakness, and new-onset diabetes. Her UFC was 9,790 mcg/24 hours, significantly elevated.

The secretion of gastrin and ACTH by a NET is relatively rare. The incidence of CS affects less than 1% of patients with ZES [2]. Its clinical presentation varies and tends to be misdiagnosed. In terms of presentation, the simultaneous occurrence of ZES and CS is usually not seen. ZES preceding CS has been reported in multiple cases. Maragliano et al. reported that, in 71% of the gastrin and ACTH-secreting pNETs, the symptoms of gastrinoma preceded those of ectopic ACTH production on an average of 33 months [8]. With the progression of the disease/liver metastasis, it is likely that some transformation in the histopathological and endocrinological nature of the pancreatic islet tumor cells occurs, and neoplastic cells acquire the ability to secrete ACTH [8]. In our patient, the symptoms of CS appeared much later than her initial symptoms of GERD and diarrhea. ACTH production is likely a sign of gastrinomas becoming malignant/metastatic, thus making a strong case for considering surgical therapy in gastrinomas that may otherwise appear to be benign. This makes it important to screen early for CS in ZES patients for early detection of this aggressive disease.

The treatment goals of patients with the secretion of gastrin and ACTH include 1) rapid control of ectopic ACTH production/adrenal cortisol over secretion; 2) treatment of the consequences of hormonal excess such as hypokalemia, high blood sugars, and acid hypersecretion; and 3) management of the tumor/metastasis secreting gastrin and ACTH.

In patients with ZES, even in the absence of tumor progression, CS can be fatal due to complications of hypercortisolemia [9]. Promptly controlling excess ACTH/cortisol production is crucial in managing the patients. If the source of ACTH overproduction is uncertain or inoperable, rescue treatment with medications or bilateral adrenalectomy is needed. The optimal therapy for the ectopic ACTH syndrome is surgical excision of the tumor, which may not be possible in metastatic tumors [7]. For patients with nonresectable tumors, hypercortisolism can be controlled with adrenal enzyme inhibitors, such as ketoconazole, metyrapone, and etomidate, and can be continued for a prolonged period. Of the 90 patients with ectopic ACTH secretion in the NIH series, 62 received medical therapy, and 20 received ketoconazole alone [7]. There was a report that the somatostatin analog effectively reduced ACTH levels in an ectopic ACTH-secreting patient [10]. Pharmacological treatment with ketoconazole and somatostatin analog, octreotide, was promptly initiated in our patient. Metyrapone or mitotane was unavailable at the pharmacy. Surgery was consulted for bilateral adrenalectomy if the cortisol levels did not rapidly improve with medical therapy. The patient responded well to ketoconazole, and reduction of the cortisol level was achieved in a short period, and it has persisted for more than a year follow-up course. The dose of ketoconazole was titrated to maintain cortisol levels in a normal range.

In gastrinoma, medical and surgical approaches are usually complementary. For most patients with ZES associated with MEN 1, medical therapy is the preferred treatment. Surgical intervention for patients with sporadic tumors without evidence of metastasis can be considered, which can reduce the risk of eventual morbidity and risk of metastatic spread of the tumor [1]. Medical management with PPIs controls hydrochloric acid hypersecretion, thus relieving symptoms and preventing complications. When PPIs cannot control gastric acid secretion, somatostatin analogs such as octreotide and lanreotide can inhibit the secretion of gastrin and cause apoptosis induction and tumor growth suppression [11-13]. Our patient was on PPI for a few years and continued it. During hospitalization, she initially received octreotide and later switched to once-a-month lanreotide (20 mg/month) to decrease ectopic hormone production and stabilize tumor cell growth.

Experience with systemic chemotherapy for metastatic gastrinoma with/without ACTH production is limited. In a recent randomized trial in pNETs, capecitabine plus temozolomide, compared with temozolomide alone, resulted in similar response rates in both groups (approximately 30%). However, the median progression-free survival was longer in the combination arm (22.7 months vs. 14.4 months, hazard ratio (HR)=0.58, p=0.023) [14]. For the capecitabine plus temozolomide group, the median overall survival was 58.7 months compared to 53.8 months for temozolomide alone (HR=0.82, p=0.42). In randomized controlled trials for pNETs, the combination therapy response rate and median progress-free survival rate are the highest reported so far [14]. Based on the trial, in advanced pNETs, capecitabine plus temozolomide chemotherapy, is the preferred regimen. Our patient is followed by the oncology team and is on the combination chemotherapy of capecitabine and temozolomide.

For metastatic disease, liver-directed therapy includes resection, radiofrequency ablation, hepatic artery embolization, cryoablation, and liver transplantation. Radioembolization with selective internal radiation therapy using Yttrium microspheres is also used, although prospective studies are yet to be completed comparing one type of embolization with another [15]. Our patient underwent Y90 therapy.

The prognosis of patients with ZES and ectopic ACTH production is poor [3]. Liver metastasis and ACTH production in gastrinomas are associated with a worse prognosis [1,3]. In one of the largest gastrinoma series of about 212 patients from NIH, Yu et al. reported that the survival rate in gastrinoma patients after ectopic ACTH secretion diagnosis averages 1.7 years [3]. In our patient, with the treatment she received so far, we did not observe disease progression after more than 18 months since the onset of CS. Mortality related to gastrinoma mostly depends upon the benign vs. malignant nature of the tumor and on the extent of the disease involvement. As earlier discussed, in patients with ZES, even in the absence of tumor progression, CS can be fatal due to complications of hypercortisolemia [9]. For pNETs, routine use of chromogranin A for diagnosis, post-treatment surveillance, or assessing therapy response is not recommended due to limited sensitivity and specificity [16].

For patients with pNETs, G1 and G2 stage with distant organ metastasis, the median survival time was 33 months, while that of G3 stage disease patients was only five months [17]. Our patient had a G2-stage tumor. The risk of recurrence could be higher in specific subgroups of patients. Patients with tumors having a Ki-67 proliferative index greater than 5%, at least in the initial period after surgery, may need more frequent surveillance. In patients with a Ki-67 proliferative index greater than 5%, as seen in our patient, Commonwealth Neuroendocrine Tumor Research Collaborative guidelines for surveillance suggest imaging frequency to be every six to 12 months for the first three years and then every one to two years for at least 10 years [18].

## Conclusions

Evaluating patients for CS should be considered when presenting with new onset or poorly controlled diabetes, weakness, and hypokalemia, even in the absence of typical stigmata. Control of excess cortisol is crucial in managing ectopic ACTH-secreting malignant tumors and can be achieved either by medications or bilateral adrenalectomy. Gastrinomas can be difficult to diagnose due to nonspecific symptoms, but early diagnosis can potentially reduce further comorbidities and improve prognosis. Somatostatin analogs can reduce hormonal secretion as well as tumor control. Capecitabine and temozolomide combination chemotherapy is the preferred regimen for malignant NETs. Treatment for patients with metastatic NETs will have to be individualized. With combination therapy of steroidogenesis inhibitors, somatostatin analogs, chemotherapy, and hepatic radioembolization, our patient remains stable at 18 months follow-up.
